# Fractionation of sulfur isotopes by *Desulfovibrio vulgaris* mutants lacking hydrogenases or type I tetraheme cytochrome *c*_3_

**DOI:** 10.3389/fmicb.2013.00171

**Published:** 2013-06-25

**Authors:** Min Sub Sim, David T. Wang, Grant M. Zane, Judy D. Wall, Tanja Bosak, Shuhei Ono

**Affiliations:** ^1^Department of Earth and Planetary Sciences, Northwestern UniversityEvanston, IL, USA; ^2^Department of Earth, Atmospheric, and Planetary Sciences, Massachusetts Institute of TechnologyCambridge, MA, USA; ^3^Department of Biochemistry, University of MissouriColumbia, MO, USA

**Keywords:** sulfate reduction, isotope effect, electron transport, hydrogen cycling, mutation

## Abstract

The sulfur isotope effect produced by sulfate reducing microbes is commonly used to trace biogeochemical cycles of sulfur and carbon in aquatic and sedimentary environments. To test the contribution of intracellular coupling between carbon and sulfur metabolisms to the overall magnitude of the sulfur isotope effect, this study compared sulfur isotope fractionations by mutants of *Desulfovibrio vulgaris* Hildenborough. We tested mutant strains lacking one or two periplasmic (Hyd, Hyn-1, Hyn-2, and Hys) or cytoplasmic hydrogenases (Ech and CooL), and a mutant lacking type I tetraheme cytochrome (TpI-*c*_3_). In batch culture, wild-type *D. vulgaris* and its hydrogenase mutants had comparable growth kinetics and produced the same sulfur isotope effects. This is consistent with the reported redundancy of hydrogenases in *D. vulgaris*. However, the TpI-*c*_3_ mutant (Δ*cycA*) exhibited slower growth and sulfate reduction rates in batch culture, and produced more H_2_ and an approximately 50% larger sulfur isotope effect, compared to the wild type. The magnitude of sulfur isotope fractionation in the CycA deletion strain, thus, increased due to the disrupted coupling of the carbon oxidation and sulfate reduction pathways. In continuous culture, wild-type *D. vulgaris* and the CycA mutant produced similar sulfur isotope effects, underscoring the influence of environmental conditions on the relative contribution of hydrogen cycling to the electron transport. The large sulfur isotope effects associated with the non-ideal stoichiometry of sulfate reduction in this study imply that simultaneous fermentation and sulfate reduction may be responsible for some of the large naturally-occurring sulfur isotope effects. Overall, mutant strains provide a powerful tool to test the effect of specific redox proteins and pathways on sulfur isotope fractionation.

## Introduction

Microbial sulfate reduction (MSR) is an anaerobic metabolism that remineralizes nearly 50% of organic matter in marine sediments (Jorgensen, 1982). Sulfate containing the light sulfur isotope (^32^S) is preferentially reduced to sulfide during this process, enriching the residual sulfate in heavy isotopes of sulfur, ^33^S, ^34^S, and ^36^S. Consequently, the isotopic compositions of various sulfur species are used to probe the coupled cycles of sulfur and carbon in nature, where the magnitude of ^34^S/^32^S fractionation varies from 0 to 77‰ (Kaplan et al., 1963; Lyons, 1997; Rudnicki et al., 2001). Laboratory studies exploring this wide range of isotope effects have related the magnitude of isotope fractionation to the influence of organic substrates (Chambers et al., 1975; Kleikemper et al., 2004; Sim et al., 2011a), sulfate (Habicht et al., 2002, 2005), iron (Sim et al., 2012), and temperature (Canfield et al., 2006; Mitchell et al., 2009). However, few studies to date have attempted to link intracellular mechanisms and enzymatic activities directly to the measured fractionation of sulfur isotopes (e.g., Farquhar et al., 2003; Mangalo et al., 2008). Fortunately, recent advances in molecular biology have enabled investigations of the biochemical basis of MSR by mutant analyses of *Desulfovibrio vulgaris* Hildenborough lacking cytoplasmic hydrogenases (Stolyar et al., 2008; Walker et al., 2009), periplasmic hydrogenases (Pohorelic et al., 2002; Goenka et al., 2005; Caffrey et al., 2007), cytochromes (Semkiw et al., 2010), and transmembrane complexes (Dolla et al., 2000; Zane et al., 2010). These advances also allow us to probe the contributions of individual enzymes and electron carriers to the sulfur isotope effect produced by MSR.

**Figure 1:**
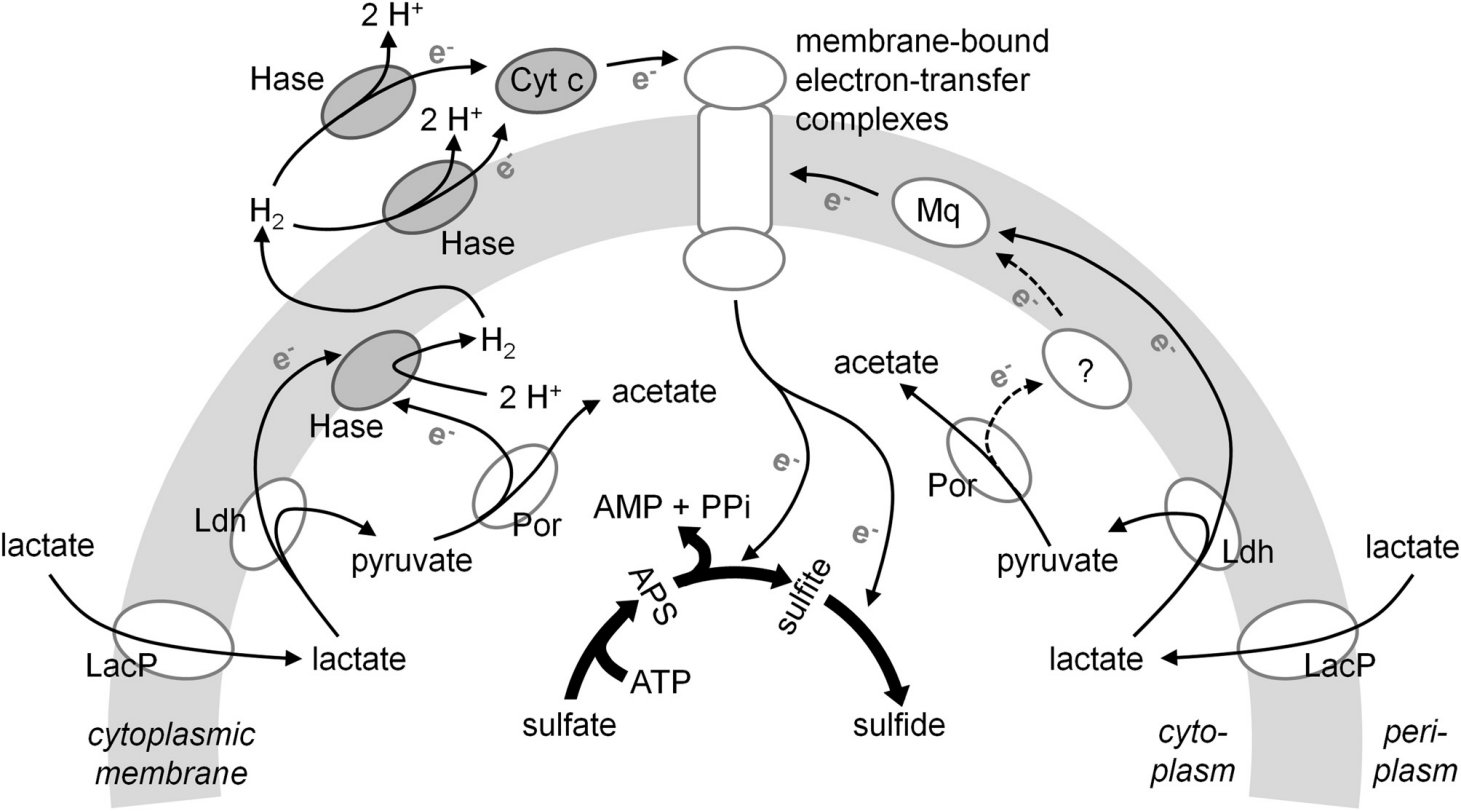
**Schematic representation of two proposed pathways for electron transport during sulfate reduction in *Desulfovibrio vulgaris* Hildenborough (Modified after Keller and Wall, 2011).** The hydrogen cycling model (Odom et al., 1981) describes the flow of reducing equivalents from the electron donor to oxidized sulfur species through hydrogen metabolism. This flow can be mediated by hydrogenases and other electron carriers, including cytochromes (Heidelberg et al., 2004). The second pathway can transfer electrons to the membrane-associated menaquinone pool (Keller and Wall, 2011). Dark gray ovals indicate enzymes and electron carriers deleted in the mutant strains used in this study. Abbreviations: LacP, lactate permease; Ldh, lactate dehydrogenase; Por, pyruvate-ferrodoxin oxidoreductase; Hase, hydrogenase; Cyt. *c*, cytochrome *c*; Mq, menaquinone pool; APS, adenosine 5′-phosphosulfate. Dashed lines and the question mark indicate currently hypothetical pathways and components.

Redox proteins that transfer electrons to the sulfate reduction pathway are likely to influence the magnitude of sulfur isotope effect, in particular when MSR is limited by electron donors (Chambers et al., 1975; Hoek et al., 2006; Sim et al., 2011a) or iron (Sim et al., 2012). At least two different electron transport pathways are known to operate in *D. vulgaris* (Noguera et al., 1998; Keller and Wall, 2011): a hydrogen cycling pathway that uses H_2_ as an intermediate electron carrier between the periplasm and the cytoplasm (Figure 1, left), and a pathway that bypasses hydrogen cycling and transfers electrons directly to the membrane-bound menaquinone pool (Figure 1, right). The hydrogen cycling pathway requires both cytoplasmic and periplasmic hydrogenases to form H_2_ in the cytoplasm and oxidize H_2_ in the periplasm. This pathway also requires cytochromes to deliver electrons from periplasmic hydrogenases to transmembrane complexes that ultimately transfer electrons to terminal reductases (Odom et al., 1981; Heidelberg et al., 2004). Consequently, the deletion of genes encoding any of these components may impair the hydrogen cycling pathway, resulting in different phenotypes. *D. vulgaris* mutants missing periplasmic hydrogenases grow more slowly compared to the wild type in lactate- or H_2_-grown batch cultures (Pohorelic et al., 2002; Caffrey et al., 2007). Some of these mutants also produce more H_2_ and CO compared to wild type at the onset of growth on lactate or pyruvate, suggesting a weaker coupling of carbon catabolism and sulfate reduction pathways (Voordouw, 2002). The mutant lacking cytoplasmic hydrogenase Ech also produces H_2_ when growing on sulfate with lactate (Stolyar et al., 2008). *D. vulgaris* mutant lacking TpI-*c*_3_ (Figure 1) cannot grow on sulfate with H_2_ or formate and grows 50% more slowly on pyruvate and sulfate compared to the wild type (Semkiw et al., 2010). These differences in growth rates and sulfate reduction rates suggest that some of these mutants also may produce different sulfur isotope effects relative to the wild type.

Here we use mutant strains of *D. vulgaris* Hildenborough to ask how disruptions in the electron transfer chain affect sulfur isotope fractionation. To the best of our knowledge, this is the first attempt to use mutant strains to study sulfur isotope effects produced by sulfate reducing microbes.

## Materials and methods

### Bacterial strains and growth medium

Various mutant strains and the corresponding parent strains of *D. vulgaris* Hildenborough were examined, including mutants lacking periplasmic hydrogenases, cytoplasmic hydrogenases, and type I tetraheme cytochrome *c*_3_ (TpI-*c*_3_) (Table 1). All mutants derived from the wild-type *D. vulgaris* lacking the 202 kb native plasmid (pDV1) were kindly provided by Dr. Gerrit Voordouw (University of Calgary, Alberta, Canada). All other mutants were described previously (Stolyar et al., 2008; Walker et al., 2009; Semkiw et al., 2010). All strains were cultured in a chemically defined, phosphate-buffered medium containing (per liter): 3 g Na_2_SO_4_, 7 g NaCl, 0.3 g Na_3_-citrate·2H_2_O, 0.32 g KH_2_PO_4_, 0.25 g K_2_HPO_4_, 1 g MgCl_2_·6H_2_O, 0.1 g KCl, 0.1 g CaCl_2_·2H_2_O, 1 mg resazurin, 1 ml of trace metal solution and 10 ml of vitamin solution. The trace metal solution contained (per liter): 1 g FeCl_2_·4H_2_O, 0.5 g MnCl_2_·4H_2_O, 0.3 g CoCl_2_·4H_2_O, 0.2 g ZnCl_2_, 0.05 g Na_2_MoO_4_·4H_2_O, 0.02 g H_3_BO_3_, 0.1 g NiSO_4_·6H_2_O, 2 mg CuCl_2_·2H_2_O, 6 mg Na_2_SeO_3_·5H_2_O, and 8 mg Na_2_WO_4_·2H_2_O. The vitamin solution contained (per liter): 2 mg biotin, 2 mg folic acid, 10 mg pyridoxine-HCl, 5 mg thiamin-HCl, 5 mg riboflavin, 5 mg nicotinic acid, 5 mg pantothenic acid, 5 mg *p*-aminobenzoic acid, and 1 mg vitamin B_12_. Titanium (III) citrate (0.1 mM) was added as a reducing agent (Zehnder and Wuhrmann, 1976; Louie and Mohn, 1999). Cultures contained either lactate (20 mM) or pyruvate (34 mM) as electron donors and carbon sources. The stoichiometric reduction of one sulfate ion requires the oxidation of two lactate or four pyruvate molecules to acetate and bicarbonate:

(1) $${\text{2CH}}_{3}\text{CH}(\text{OH}){\text{COO}}^{-}+{\text{SO}}_{4}^{2-}\to {\text{2CH}}_{3}{\text{COO}}^{-}+{\text{2HCO}}_{3}^{-}+{\text{HS}}^{-}+{\text{H}}^{+}$$

(2) $${\text{4CH}}_{3}{\text{COCOO}}^{-}+{\text{SO}}_{4}^{2-}+{\text{4H}}_{2}\text{O}\to {\text{4CH}}_{3}{\text{COO}}^{-}+{\text{4HCO}}_{3}^{-}+{\text{HS}}^{-}+{\text{3H}}^{+}$$

**Table 1:** ***Desulfovibrio vulgaris* strains used in this study**.

**Strain**	**Parent strain**	**Description**	**References**
WT	NA^a^	Wild-type strain, *Desulfovibrio vulgaris* Hildenborough	ATCC 29579
JW375	NA^a^	A spontaneously nalidixic acid-resistant strain lacking pDV1	
JW710	WT	Wild-type *D. vulgaris* strain deleted of the *upp* gene	Keller et al. (2009)
WT Δ*pDV1*	NA^a^	Wild-type strain lacking pDV1	
JW380	WT	Deletion of DVU0434 (Δ*echA*), cytoplasmic [NiFe] hydrogenase	Stolyar et al. (2008)
JW9087	WT	Deletion of DVU3171 (Δ*cycA*), Type I tetraheme cytochrome *c*_3_	Semkiw et al. (2010)
JW3040	JW375	Transposon interruption of DVU2288 (Δ*cooL*), cytoplasmic [NiFe] hydrogenase	Walker et al. (2009)
JW9135^b^	JW710	Deletion of DVU1769-1770 (Δ*hydAB*), periplasmic [Fe] hydrogenase	
JW9137^b^	JW710	Deletion of DVU1917-1918 (Δ*hysBA*), periplasmic [NiFeSe] hydrogenase	
JW9141^b^	JW710	Deletion of DVU1921-1922 (Δ*hynBA-1*), periplasmic [NiFe] hydrogenase	
JW9143^b^	JW710	Deletion of DVU2525-2526 (Δ*hynBA-2*), periplasmic [NiFe] hydrogenase	
Hyd100	WT Δ*pDV1*	Deletion of DVU1769-1770 (Δ*hydAB*), periplasmic [Fe] hydrogenase	Pohorelic et al. (2002)
Hys100	WT Δ*pDV1*	Deletion of DVU1917-1918 (Δ*hysBA*), periplasmic [NiFeSe] hydrogenase	Caffrey et al. (2007)
NiFe100	WT Δ*pDV1*	Deletion of DVU1921-1922 (Δ*hynBA-1*), periplasmic [NiFe] hydrogenase	Goenka et al. (2005)
NiFe200	WT Δ*pDV1*	Deletion of DVU2525-2526 (Δ*hynBA-2*), periplasmic [NiFe] hydrogenase	Caffrey (2008)
HydHyn1	WT Δ*pDV1*	Double mutant, constructed by introducing the hyn-1 mutation into Hyd100	Caffrey et al. (2007)

a NA, not applicable.

b Detailed description of each mutant can be seen on the following website (http://desulfovibriomaps.biochem.missouri.edu/mutants/).

The medium was flushed with N_2_ gas, the pH was adjusted to 7.5 with NaOH, and the adjusted medium was sterilized by autoclaving. Filter-sterilized anaerobic solutions of titanium citrate, vitamins, and Ca and Mg were added after autoclaving. The final pH of the medium was between 7 and 7.5.

### Culture experiments

All strains in batch cultures were incubated at 37°C. Sterile medium (20 ml in a 25 ml bottle) was inoculated with cells that had been washed three times by centrifugation and resuspension in anaerobic fresh medium to reduce the carryover of sulfide. Cell growth and sulfate reduction rates were monitored three times a day. For measurements of sulfate and sulfide, a 200 μl culture sample was mixed with 1 ml of 0.05 M zinc acetate solution and stored at 4°C to fix sulfide as zinc sulfide. Sulfide and sulfate concentrations were measured by a modified methylene blue assay (Cline, 1969) and by a turbidimetric assay (Lundquist et al., 1980), respectively. The uncertainty of these measurements was ±5% for sulfide and ±10% for sulfate, respectively. Samples used to analyze organic acids were filter sterilized through 0.2 μm syringe filters (Whatman, Clifton, NJ, USA) and stored at −20°C. Pyruvate and acetate were quantified enzymatically with K-PYRUV and K-ACET assay kits (Megazyme, Wicklow, Ireland), respectively. The concentration of formate was determined with the EnzyLITE™ assay kit EZ-0035 (Assay Biotechnology Company, Sunnyvale, CA, USA). Analyses of organic acids were subject to ±10% error. Cell growth was monitored by measuring the optical density at 630 nm on the Synergy2 Biotek microplate reader, (BioTek, Winooski, VT, USA) and by microscopic counts of cells stained by SYTOX-Green nucleic acid stain (Invitrogen S7020, Paisley, UK) with a Zeiss Axio Imager M1 epifluorescence microscope (Carl Zeiss, Thornwood, NY, USA). At the end of the incubation, a 3-ml aliquot of 1 M Zn-acetate was added to the bottle to terminate microbial activity and to precipitate dissolved sulfide as zinc sulfide (Detmers et al., 2001). From those bottles, 300 μl of headspace gas were transferred by a gas-tight syringe to a gas chromatograph (Shimadzu GC-2014) equipped with TCD-methanizer-FID and a packed column (1/8″ outer diameter, 4.6 m length, Carboxen-1000 60/80 mesh, Supelco) to measure H_2_ and CO. Procedural reproducibility of these measurements was 8%, as determined by replicate sample analyses. Average cell specific sulfate reduction rates (csSRRs) in batch cultures were calculated for growing cells according to Eq. (3) as previously described (Sim et al., 2012):

(3) $$\text{csSRR}=\frac{{\left[{\text{H}}_{2}\text{S}\right]}_{N}-{\left[{\text{H}}_{2}\text{S}\right]}_{1}}{\sum_{n=1}^{N-1}\frac{C_{n}+C_{n\;+1}}{2}\cdot (t_{n\;+1}-t_{n})}$$

where [H_2_S]_1_ and [H_2_S]_*N*_ are sulfide concentrations at the first detection of measurable sulfide and at the last sampling time before the cessation of growth, *t*_*n*_ is the time of sampling, and *C*_*n*_ is the cell density at each sampling point. Terms in the denominator give the time-weighted average for cell numbers. Equation (3) applies both to linear and exponential growth (Sim et al., 2012).

Continuous cultures of the wild type and the TpI-*c*_3_ mutant (Δ*cycA*) were grown in a reactor that consisted of a 500 ml water-jacketed flask with ports for sampling, gas outlet, and medium entry and discharge. The medium containing 20 mM pyruvate and 21 mM sulfate was prepared as described above, but was buffered by bicarbonate instead of phosphate, and reduced by 5 mM sodium ascorbate and 0.01 mM titanium citrate instead of 0.1 mM titanium citrate to prevent the precipitation of titanium hydroxide. The medium was pumped into and out of the reactor by a peristaltic pump (Gilson Minipuls3, Villiers le Bel, France) at a dilution rate of 0.02 h^−1^. Both the reactor and the fresh medium were gassed with humidified 80% N_2_/20% CO_2_ gas at a flow rate of 65 ± 2 ml/min to maintain the anoxic conditions and buffer the pH. This gas also purged sulfide produced in the reactor and delivered it to a sulfide trap containing 0.18 M zinc acetate solution. Cell density was monitored twice a day and the culture was assumed to be in a steady state when the cell density remained constant (within ±5%) for 2 successive days. The concentrations and δ^34^S of sulfate, concentrations of pyruvate and acetate, and cell numbers were measured in samples of the discharge flow. The concentrations and δ^34^S of sulfide and the partial pressure of H_2_ were measured in samples of the gas outlet stream. The temperature of the reactor was maintained at 37 ± 1°C.

### Isotopic measurements

Zinc sulfide was recovered from fixed samples by centrifugation. The collected ZnS (ca. 10 μmoles) was resuspended in 400 μl of distilled water, mixed with 500 μl of AgNO_3_ stock solution (1.7 g of AgNO_3_ in 100 ml of 0.1 M HNO_3_), and incubated at 65°C overnight to precipitate Ag_2_S. The resulting Ag_2_S was washed three times with distilled water, and dried at 80°C for 1 day. Sulfate in the fresh medium (3 ml) was reduced to sulfide by a reaction with 30 ml of the reducing agent (mixture of HI, H_3_PO_2_ and HCl, Thode et al., 1961), and boiled and purged with N_2_ for 2 h. Volatile products were passed through a condenser and a trap containing distilled water, and sulfide was collected in a Zn-acetate trap. ZnS was converted to Ag_2_S as described above. The Ag_2_S samples were allowed to react with an excess of fluorine gas for more than 5 h at 300°C, and the produced SF_6_ was purified by gas chromatography. Purified SF_6_ was transferred into an isotope-ratio mass spectrometer for sulfur isotope measurements in the dual inlet mode (Ono et al., 2006).

The isotope fractionation factor (^34^α) in batch cultures was calculated from the measured isotopic compositions of the initial sulfate and the produced sulfide following the modified Rayleigh distillation equation (Mariotti et al., 1981):

(4) $${}^{34}\alpha =\frac{1}{\ln f_{r}}\ln\left(1-(1-f_{r})\frac{\delta^{34}S_{\text{HS}}+1000}{1000}\right)$$

where *f*_*r*_ is the fraction of the remaining sulfate, and δ^34^*S*_HS_ is the isotopic composition of sulfide normalized to that of the starting sulfate. The analytical uncertainty of sulfur isotope measurements is 0.2‰. Isotope fractionation factors in continuous cultures at the steady state were calculated as:

(5) $${}^{34}\alpha =\frac{\delta^{34}S_{\text{HS}}+1000}{\delta^{34}S_{{\text{SO}}_{4}}+1000}$$

The isotope enrichment factor is defined as:

(6) $${}^{34}\varepsilon =1000\cdot (1{-}^{34}\alpha )$$

According to this definition, positive values represent the depletion of heavy isotopes in the product.

## Results

### Growth of *D. vulgaris* mutants in batch culture

**Figure 2:**
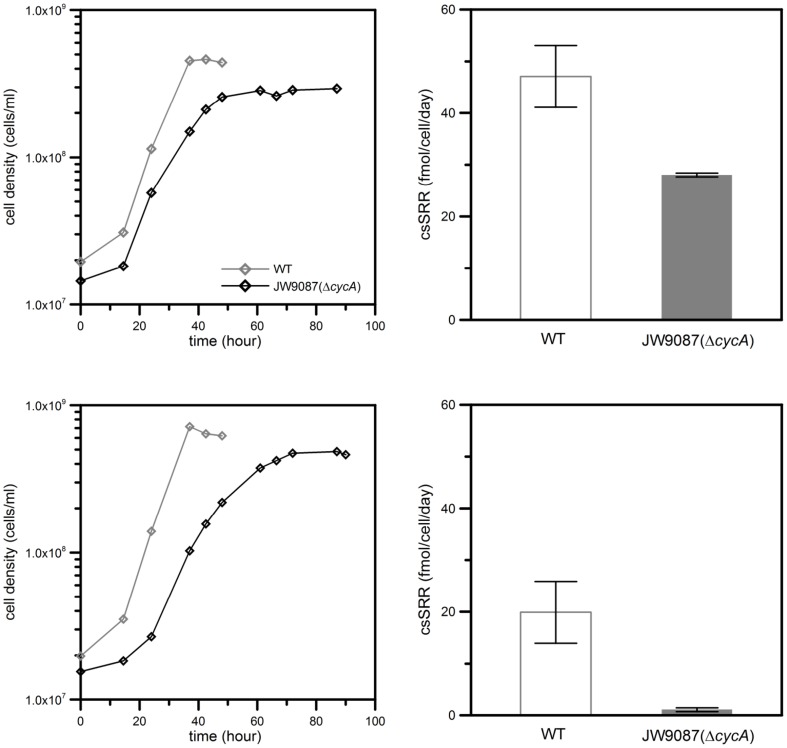
**Growth (left) and cell specific sulfate reduction rate (csSRR) (right) of wild-type *Desulfovibrio vulgaris* Hildenborough and CycA mutant grown on lactate (upper) and pyruvate (lower).** Growth curves are representative of two or more independent experiments. Errors for csSRR are given as the standard deviation of the mean.

Wild-type *D. vulgaris* and all tested mutants were capable of growth on lactate or pyruvate as the sole carbon sources and reductants. All cytoplasmic hydrogenase mutant strains grew at rates similar to their parent strains (data not shown, Stolyar et al., 2008). The same was true for periplasmic hydrogenase mutants under our experimental conditions (data not shown), although previous studies reported somewhat lower growth rates and yields relative to the wild type (Voordouw, 2002; Caffrey et al., 2007). In contrast to the hydrogenase mutants, the cell yield and growth rate of the CycA mutant were lower than those of the wild type both in lactate- and pyruvate-grown cultures (Figure 2). With lactate as the electron donor, the wild-type and CycA mutant strains reduced sulfate at cell specific sulfate reduction rates (csSRR) of 47.1 fmol/cell/day and 28.0 fmol/cell/day, respectively (Table 2). When the reaction proceeded to completion (i.e., all lactate was consumed), both the wild type and the CycA mutant produced twice as much acetate as sulfide, according to the expected stoichiometry (Eq. 1, Table 2). However, the observed concentrations of products at the end of growth on pyruvate differed from the expected ones (Eq. 2). Both wild-type and CycA cultures consumed all pyruvate, but reduced less sulfate than predicted by the ideal reaction stoichiometry (Table 2, Eq. 2). In particular, the CycA mutant generated only 0.9 mM sulfide, instead of the expected 8.4 mM concentration and respired sulfate 20 times slower than the wild type. This is consistent with a previous mutant analysis of *Desulfovibrio desulfuricans* G20 (now *Desulfovibrio alaskensis*) lacking TpI-*c*_3_ (Rapp-Giles et al., 2000).

**Table 2:** **Utilization of substrates and compounds produced by wild-type *Desulfovibrio vulgaris* Hildenborough and CycA mutant at the end of batch culture experiments**.

**Strain, genotype**	**Initial culture medium**	**Remaining substrate (mM)**		**Product formed**			
		**Organic acid**	**Sulfate^a^**	**Sulfide (mM)^a^**	**Acetate (mM)^a^**	**Formate (mM)**	**H_2_ (μmole)**
WT	20 mM lactate, 21 mM sulfate	ND^b^	10.3	10.0	17.1	0.1	0.1
	34 mM pyruvate, 21 mM sulfate	0.6	15.7 (12.7)	6.2 (8.3)	31.2 (33.4)	0.2	0.1
JW9087, Δ*cycA*	20 mM lactate, 21 mM sulfate	ND^b^	12.0	8.3	18.5	1.6	79.5
	34 mM pyruvate, 21 mM sulfate	0.4	19.2 (12.6)	0.9 (8.4)	26.1 (33.6)	BDL^c^	84.0

a Numbers in parentheses indicate the expected concentrations of sulfate and sulfide according to reaction stoichiometries for Eq. (2).

b ND, not determined

c BDL, below detection limit.

Because the deletion of *cycA* reduced the respiration rate and growth yield in batch culture, we hypothesized that some of the reducing equivalents may be released from the cell as H_2_ or CO, instead of being used to reduce sulfate. Previous studies reported the production of H_2_ and CO from periplasmic and cytoplasmic hydrogenase mutants (Voordouw, 2002; Stolyar et al., 2008), but not from a strain deleted for *cycA*. Therefore, we measured the concentrations of H_2_ and CO in the headspace of early stationary phase cultures of the CycA mutant (Table 2). The wild type grown on lactate or pyruvate produced trace amounts of hydrogen (0.1 μmol), but almost 1000 times higher hydrogen concentration was present in the headspace of CycA cultures (Table 2). CO concentrations in the headspace of any early stationary phase cultures were equal to the blank value (0.04 μM). Albeit high, the H_2_ concentration in the headspace of CycA cultures can account for only about 10% of the electrons released by the oxidation of lactate or pyruvate. Therefore, the full reaction stoichiometry of pyruvate metabolism by the CycA deletion mutant remains to be characterized in the future.

### Fractionation of sulfur isotopes in batch cultures

**Figure 3:**
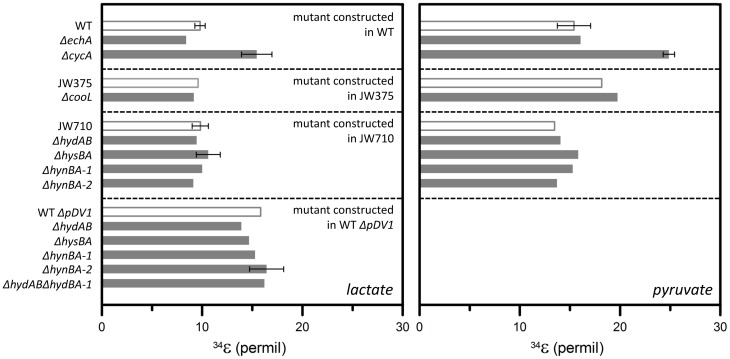
**Sulfur isotope effects produced by *Desulfovibrio vulgaris* Hildenborough and several mutants grown on lactate (left) and pyruvate (right).** Mutants (gray bars) are grouped with their respective parent strains (white bars). When multiple experiments were performed with independent cultures, horizontal error bars indicate standard deviations.

In keeping with the slower growth and sulfate reduction rates of the CycA mutant, sulfide in the cultures of this mutant was more depleted in the heavy isotope (^34^S) than sulfide produced by any other strain (Figure 3). In batch experiments, the CycA mutants grown on lactate or pyruvate fractionated ^34^S/^32^S at 15.5 and 24.9‰, respectively. This exceeded the corresponding fractionations by the wild type, 9.8 and 15.4‰, respectively. Calculated enrichment factors (^34^ε) in batch cultures of mutants other than CycA ranged from 8.4 to 16.4‰ in lactate-grown cultures, and from 13.5 to 19.8‰ in pyruvate-grown cultures (Figure 3). All strains in pyruvate-grown cultures fractionated ^34^S/^32^S by 4‰ more relative to the values in lactate-grown cultures (Figure 3). A similar trend between lactate- and pyruvate-grown cells was previously reported in cultures of a different *Desulfovibrio* species (Sim et al., 2011a).

Hydrogenase mutant strains also yielded a range of sulfur isotope fractionations (Figure 3, Table 1). The magnitudes of these fractionations, however, reflected the use of different parent strains in the construction of hydrogenase mutants, rather than the deletion of any specific hydrogenase gene. For example, JW375, the parent strain for the CooL mutant, fractionated ^34^S/^32^S 5‰ more than JW710, the parent strain for four different periplasmic hydrogenase mutants, when grown on pyruvate (Figure 3). The difference among sulfur isotope effects of the two parent strains can fully account for any differences in the sulfur isotope effects produced by the CooL mutant and other periplasmic hydrogenase mutants (Figure 3). Furthermore, mutants lacking the same periplasmic hydrogenase gene but constructed in different genetic backgrounds (JW710, WT Δ*pDV1*) produced sulfur isotope effects that differed by as much as 6‰, but so did their respective parent strains (Figure 3, Table 1). Presently, factors responsible for these reproducible differences among the sulfur isotope effects of various parent strains remain unclear.

### Growth and sulfur isotope effect in continuous culture of the CYCA mutant

To test the effect of growth conditions, including the large concentrations of organic carbon present in batch cultures, on the metabolism and sulfur isotope effect produced by the CycA mutant, we grew this mutant on pyruvate in continuous culture. Continuous cultures of the wild type and the mutant contained less than 300 μM pyruvate in the outflow (Table 3), confirming that the growth was limited by this organic acid. Nevertheless, the wild-type and CycA deletion strains produced only 2.3 mM and 1.1 mM sulfide per 20 mM pyruvate consumed, respectively, i.e., less than the 5 mM predicted for the incomplete oxidation of pyruvate into acetate and CO_2_ (Eq. 2). Thus, fewer than half of the electrons produced by the incomplete oxidation of pyruvate were used to reduce sulfate. The production of H_2_ can account for about 70% of the missing electrons from the CycA deletion strain, because this mutant generated H_2_ at the rate of 41.5 fmol/cell/day. In contrast, H_2_ production rate was only 0.03 fmol/cell/day in the wild type (Table 3). Although the wild type reduced twenty times more sulfate per cell per unit time compared to the CycA mutant in batch cultures (Figure 2), this difference was smaller in continuous culture, where the wild type csSRR was only about two times larger than that of the CycA mutant (Table 3). Not only were the csSRRs in continuous cultures of the wild type and the CycA mutant more comparable, but the two also produced similar sulfur isotope effects under these conditions: WT: 35.5‰ and Δ*cycA*: 32.4‰ (Figure 3, Table 3). Notably, these fractionations by the wild type and the mutant strain in continuous cultures exceeded the corresponding fractionations in batch cultures by 20‰ and 7‰, respectively.

**Table 3:** **Metabolites and sulfur isotope fractionation in continuous cultures of wild-type *Desulfovibrio vulgaris* Hildenborough and CycA mutant**.

**Initial culture medium**	**Strain, genotype**	**Concentration in culture medium**					**Cell specific consumption/production rate (fmol/cell/day)**				**^34^ε (‰)**
		**Cell density (10^8^ cells/ml)**	**Pyruvate (mM)**	**Sulfate (mM)**	**Sulfide (mM)**	**Acetate (mM)**	**Pyruvate**	**Sulfide**	**Acetate**	**H_2_**	
20 mM pyruvate	WT	1.2	<0.1	19.7	2.3	10.5	78.4	8.9	41.2	0.03	35.5
21 mM sulfate	JW9087, Δ*cycA*	1.6	0.3	20.4	1.1	18.5	71.9	3.9	67.4	41.5	32.4

## Discussion

The use of wild-type *D. vulgaris* and its mutants enabled us to test how electron transfer proteins outside the sulfate reduction pathway influence the magnitude of sulfur isotope fractionation. The largest effect of a gene deletion on sulfur isotope fractionation was measured in batch cultures of the mutant lacking the type I tetraheme cytochrome *c*_3_ (TpI-*c*_3_) because of a deletion of the encoding gene, *cycA*. Unlike the CycA mutant, all hydrogenase mutants and their parent strains produced the same fractionations under our growth conditions (Figure 3). *D. vulgaris* contains at least two cytoplasmic and four periplasmic hydrogenases (Keller and Wall, 2011) and these hydrogenases are thought to have different affinitities for H_2_ (Rabus et al., 2006). In batch culture experiments, where the microbial growth occurs in the presence of abundant electron donor during much of the exponential phase, these multiple hydrogenases may be redundant (Caffrey et al., 2007). This redundancy can also explain the similarity of sulfur isotope effects produced in batch cultures of various mutants under our experimental conditions.

### Effects of TpI-*c*_3_ deletion in batch and continuous cultures

TpI-*c*_3_ has been proposed to shuttle electrons from periplasmic hydrogenases to transmembrane electron transport complexes, which then transport electrons to cytoplasmic terminal reductases (Heidelberg et al., 2004; Semkiw et al., 2010) (Figure 1). Consequently, TpI-*c*_3_ should be involved in the hydrogen cycling pathway. As an alternative to H_2_ cycling, electrons may be transported through membrane-bound menaquinone pool (Figure 1; Noguera et al., 1998; Keller and Wall, 2011). In batch cultures, TpI-*c*_3_ appears to be necessary for the efficient coupling of sulfate reduction and lactate and pyruvate oxidation by the hydrogen cycling pathway because the CycA mutant exhibited a lower csSRR and produced H_2_ faster than the wild type. The deletion of *cycA* had a stronger phenotype, including slower growth and respiration rates, in batch cultures grown on pyruvate relative to lactate, even though pyruvate is generated by the oxidation of lactate (Rabus et al., 2006). In batch cultures grown on lactate, the intracellular pyruvate concentration was suggested to be in the range of 0.1 mM (Pankhania et al., 1988), while high concentrations of pyruvate were present until the end of the exponential growth in pyruvate batch cultures. We thus infer that hydrogen cycling tends to be more important at high concentrations of pyruvate. Consistently, in pyruvate-limited continuous cultures, where the concentrations of pyruvate are one to two orders of magnitude lower (Table 3), the differences in csSRRs between the wild type and the CycA mutant were 10 times smaller than the differences in csSRRs in batch cultures. We hypothesize that the availability of pyruvate and subsequent changes in the intracellular redox state play critical roles in determining the pathway of electron transport. In particular, mM concentrations of pyruvate may hinder the electron transport through the menaquinone pools, which bypass TpI-*c*_3_ (Figure 1). This observation, derived from comparisons of the CycA deletion strain and the wild type in batch and continuous cultures, exemplifies the potential of growth conditions to influence organismal physiology and control the relative contributions of different electron transfer pathways during sulfate reduction.

### Sulfur isotope fractionation and electron flow to the sulfate reduction pathway

The present model of sulfur isotope fractionation during MSR has focused mainly on the enzymes that are directly involved in the transport and redox transformation of sulfur species during sulfate reduction (Rees, 1973; Farquhar et al., 2003; Bradley et al., 2011). MSR pathway is an eight electron transfer reaction carried out by several enzymatic steps, some (or all) of them operating in a reversible manner (e.g., Peck, 1961; Trudinger and Chambers, 1973). Rees (1973), Farquhar et al. (2003), Brunner and Bernasconi (2005) developed a model of sulfur isotope fractionations based on this pathway. These models ascribe the overall isotope effect to the ratio between forward and backward fluxes at each enzymatic step (reversibility), predicting maximum fractionation when the reversibility approaches unity. All these models highlight the roles of enzymes in the MSR pathway, and make much more implicit assumptions about the influence of other, unspecified redox proteins that supply electrons and ATP to the MSR pathway, although electron donors need to be oxidized and electrons transferred to the terminal reductases if MSR is to get rid of electrons under anaerobic conditions. Consequently, any deficiency in the electron transport system should slow down the reductive (forward) reactions, increase the reversibility, and, in turn, increase the overall sulfur isotope fractionation during MSR. The slower csSRR of the CycA deletion strain and the larger sulfur isotope effect produced by this mutant in batch culture support this prediction. The use of the CycA-deficient mutant also provides the first experimental link between a specific component of electron transfer chain and the magnitude of sulfur isotope effect.

### Environmental and geological implications

Trends observed in studies of a mutant strain under laboratory conditions can inform interpretations of sulfur isotope data in modern and past environments. First, although multiheme cytochromes *c* are widespread among sulfate reducing microbes (Postgate, 1956; Pereira et al., 2011), these cytochromes are absent from several sequenced species of sulfate reducers, including Gram-positive *Desulfotomaculum acetoxidans* and *Desulfotomaculum reducens* and the archaeon *Caldivirge maquilingensis* (Pereira et al., 2011). Therefore, organisms lacking *c*-type cytochromes may not rely on hydrogen cycling for the delivery of electrons to the sulfate reduction pathway. The sharp contrast in sulfur isotope fractionation between the CycA mutant and the wild type suggests that species lacking *c*-type cytochromes and other components of the classical hydrogen cycling pathway (Pereira et al., 2011) may produce larger fractionations than sulfate reducing microbes that cycle hydrogen. The comparison of batch and continuous culture experiments in this study also shows that environmental conditions can strongly influence the relative contribution of different electron transport pathways in sulfate reducing microbes (Figure 1; e.g., Noguera et al., 1998), and affect sulfur isotope fractionation. For example, intracellular levels of TpI-*c*_3_ may depend on environmental factors such as iron availability. Iron, when limited, impairs the synthesis of cytochrome *c* in sulfate reducing bacteria (Postgate, 1956; Sim et al., 2012) and increases the sulfur isotope fractionation (Sim et al., 2012).

Interestingly, whenever *D. vulgaris* or CycA mutants produced sulfur isotope effects larger than 20‰, these effects were associated with the production of no more than 2.3 ± 0.1 mM sulfide per 20 ± 2 mM of pyruvate consumed, as opposed to the ideal reaction stoichiometry of 5 mM sulfide to 20 mM pyruvate (Eq. 2). Because the mass balance between sulfide and sulfate was maintained throughout the experiment, the observed stoichiometry suggests that some energy for growth is derived from pyruvate fermentation, and that fewer than half of electrons derived from pyruvate oxidation are used to reduce sulfate. Simultaneous sulfate reduction and fermentation have also been reported in wild-type cultures of other sulfate reducing bacteria (Sass et al., 2002; Sim et al., 2011b). The model of sulfur isotope fractionation attributes larger sulfur isotope effects to an increase in the reversibility of the MSR pathway (Rees, 1973; Brunner and Bernasconi, 2005). As suggested by our results, larger rates of backward fluxes in the MSR pathway can be expected if electrons are diverted from the respiratory chain toward fermentation. Fermentation occurring at the same time as sulfate reduction may also affect the fractionation of sulfur isotopes by altering the cellular energy budget. If the generation of ATP in a growing culture depends exclusively on sulfate respiration, a minimum csSRR is required for the maintenance energy (Pirt, 1965). Thus, the reversibility of MSR, which correlates strongly with csSRR (Sim et al., 2011a), may not approach its theoretical maximum. In contrast, when sulfate reduction occurs simultaneously with fermentation, fermentation may be able to provide a part of the maintenance energy, allowing slower csSRR and leading to larger ^34^ε values. This hypothesis may explain why the largest enrichment factors obtained in this and previous studies (Sim et al., 2011b) are associated with mixed metabolisms. As many sulfate-reducing microbes are also facultative fermenters (Rabus et al., 2006), fermentation by SRMs in natural habitats and sulfur isotope signatures produced in such communities deserves further investigation.

This is the first proof-of-concept study with mutant strains that examines the effect of specific enzymatic reactions on the overall sulfur isotope effect. Although this work explores only a small number of mutant strains, future studies may investigate sulfur isotope effects in cultures of mutants lacking other key components of the electron transfer chain, such as the Hmc and Qmo complexes (Dolla et al., 2000; Zane et al., 2010). This approach can be also expanded to different species of sulfate reducing bacteria (Rapp-Giles et al., 2000; Casalot et al., 2002) and to other reactions contributing to MSR. For example, mutants lacking sulfate permeases and enzymes containing various metal cofactors may help elucidate the effects of varying sulfate and trace metal concentrations on the fractionation of sulfur isotopes in natural settings. Such studies may improve the understanding of chemical factors that influenced the magnitude of microbial sulfur isotope effects through geologic time.

### Conflict of interest statement

The authors declare that the research was conducted in the absence of any commercial or financial relationships that could be construed as a potential conflict of interest.
